# Identification of genetic loci for powdery mildew resistance in common wheat

**DOI:** 10.3389/fpls.2024.1443239

**Published:** 2024-10-09

**Authors:** Xia Liu, Xiaoqing Zhang, Xianghai Meng, Peng Liu, Menglin Lei, Hui Jin, Yanzhen Wang, Yirong Jin, Guoqing Cui, Zhixin Mu, Jindong Liu, Xiaoyun Jia

**Affiliations:** ^1^ College of Agriculture, Shanxi Agricultural University, Taigu, China; ^2^ Center for Agricultural Genetic Resources Research, Shanxi Agricultural University/Key Laboratory of Gene Resources and Germplasm Enhancement, Ministry of Agriculture and Rural Affairs/Key Laboratory of Crop Genetics and Molecular Improvement of Shanxi Province, Taiyuan, China; ^3^ National Agricultural Technology Extension Service Center of the Ministry, Agriculture and Rural Affairs, Beijing, China; ^4^ Dryland Farming Institute, Hebei Academy of Agricultural and Forestry Sciences, Hengshui, China; ^5^ Wheat Research Institute, Dezhou Academy of Agricultural Sciences, Dezhou, China; ^6^ Institute of Forage and Grassland Sciences, Heilongjiang Academy of Agricultural Sciences, Harbin, China; ^7^ Institute of Crop Sciences, National Wheat Improvement Centre, Chinese Academy of Agricultural Sciences (CAAS), Beijing, China

**Keywords:** 90K SNP array, KASP, powdery mildew, *Triticum aestivum*, resistance

## Abstract

Powdery mildew (PM) poses an extreme threat to wheat yields and quality. In this study, 262 recombinant inbred lines (RILs) of Doumai and Shi 4185 cross were used to map PM resistance genes across four environments. High-density genetic linkage map of the Doumai/Shi 4185 RIL population was constructed using the wheat Illumina iSelect 90K single-nucleotide polymorphism (SNP) array. In total, four stable quantitative trait loci (QTLs) for PM resistance, *QPm.caas-2AS*, *QPm.caas-4AS*, *QPm.caas-4BL*, and *QPm.caas-6BS*, were detected and explained 5.6%–15.6% of the phenotypic variances. Doumai contributed all the resistance alleles of *QPm.caas-2AS*, *QPm.caas-4AS*, *QPm.caas-4BL*, and *QPm.caas-6BS*. Among these, *QPm.caas-4AS* and *QPm.caas-6BS* overlapped with the previously reported loci, whereas *QPm.caas-2AS* and *QPm.caas-4BL* are potentially novel. In addition, six high-confidence genes encoding the NBS-LRR-like resistance protein, disease resistance protein family, and calcium/calmodulin-dependent serine/threonine-kinase were selected as the candidate genes for PM resistance. Three kompetitive allele-specific PCR (KASP) markers, *Kasp_PMR_2AS* for *QPm.caas-2AS*, *Kasp_PMR_4BL* for *QPm.caas-4BL*, and *Kasp_PMR_6BS* for *QPm.caas-6BS*, were developed, and their genetic effects were validated in a natural population including 100 cultivars. These findings will offer valuable QTLs and available KASP markers to enhance wheat marker-assisted breeding for PM resistance.

## Highlights

- Four QTLs for powdery mildew resistance were identified in the Doumai/Shi 4185 wheat RIL population. KASP markers for *QPm.caas-2AS* and *QPm.caas-6BS* were validated in a panel of wheat cultivars.

## Introduction

Common wheat (*Triticum aestivum* L.) is one of the most crucial food crops globally, and it provides nearly 20% of human caloric intake and fulfills 25% of protein requirements. However, its productivity suffers from various biotic and abiotic threats. Powdery mildew (PM), caused by the fungal pathogen *Blumeria graminis* f. sp. *tritici* (Bgt), is a particularly devastating destroy affecting leaves, spikelets, and awns. Since the 1970s, PM has extensively spread across nearly all major wheat cropping areas in mainland China (Kang et al., 2020a; Wang et al., 2023). Annual losses due to PM have surpassed eight million hectares worldwide in 2020s (Jia et al., 2020; Kang et al., 2020b; Wang et al., 2023). Although PM can be controlled by fungicides, it incurs a huge cost and causes environmental pollution. Breeding and employing PM-resistant cultivars are the most economical, environmentally friendly, and effective approaches to decrease economic losses (Ma et al., 1994; Kang et al., 2020a).

PM resistance can be categorized into all-stage resistance (ASR) and adult-plant resistance (APR). Typically, ASR is governed by major genes and provides immune or highly resistant responses to specific pathogen races throughout the entire growth cycle. Conversely, APR, generally mediated by minor genes, is less durable and can be easily overcome by emerging pathogen races. However, APR is often effective against all races of Bgt. The integration of four to five APR genes can result in a durable and high level of resistance (Li et al., 2016; Jia et al., 2020; Wu et al., 2021; Bapela et al., 2023). Due to its race non-specificity and longevity, APR has gained increasing importance in wheat breeding programs. To date, nearly 100 PM resistance alleles have been identified at over 60 loci (*Pm1* to *Pm68*), and over 150 PM resistance loci are distributed across all 21 wheat chromosomes (Shah et al., 2018; Kang et al., 2020a; Simeone et al., 2020; Zhang et al., 2021; Wang et al., 2023). Although a massive number of loci for powdery mildew resistance have been reported, some PM resistance genes have negative pleiotropic effects. For example, *Pm8* caused wheat quality reduction, whereas *Pm16* led to a 15% yield loss (Summers and Brown, 2013; Li et al., 2020). Although there is already a substantial amount of research on powdery mildew, some studies use traditional simple sequence repeat (SSR) or diversity array technology (DArT) markers, which are less suitable for the current breeding. Thus, novel PM resistance genes were needed to accelerate wheat PM resistance breeding.

With the development of genotyping techniques (Liu et al., 2016; Liu J. et al., 2017; Liu et al., 2019), unearthing quantitative trait loci (QTLs) for complex traits has been accelerated by utilizing the single-nucleotide polymorphism (SNP) array, such as grain quality, yield, and disease resistance-related traits (Liu J. et al., 2017; Jia et al., 2020; Kang et al., 2020a, 2020b; He et al., 2021). Tightly linked or significantly associated SNPs can be transformed into kompetitive allele-specific PCR (KASP) markers and incorporated into marker-assisted selection (MAS) breeding (Rasheed et al., 2016; Kaur et al., 2020). Doumai is a landrace with APR to PM, whereas Shi 4185 is a widely grown higher-yield winter wheat cultivar with high susceptibility to PM. A total of 262 F_2:6_ recombinant inbred lines (RILs) derived from Doumai/Shi 4185 were used for PM resistance evaluation and novel locus identification. The objectives of this study were to 1) identify PM resistance loci and tightly linked SNP markers, 2) excavate candidate genes for further study, and 3) develop available KASP markers for wheat PM resistance breeding.

## Materials and methods

### Plant materials and phenotyping of powdery mildew

The 262 F_2:6_ RILs derived from Doumai/Shi 4185 were used for evaluating PM reactions. Additionally, a diverse panel with 100 cultivars originating from the Yellow-Huai Wheat Region were used to validate the effectiveness of developed KASP markers. All 262 F_2:6_ RILs and their parents were evaluated for PM reaction in Dezhou of Shandong and Shijiazhuang of Hebei during the 2020–2021 and 2021–2022 cropping seasons. The 100 wheat cultivars were planted in Gaoyi and Shijiazhuang of Hebei and Beijing during the 2020–2021 cropping season.

All RILs and 100 cultivars were planted in a randomized complete block design with three replicates. Each plot consisted of a single 1-m row, spaced 30 cm apart, containing approximately 50 plants. The highly susceptible cultivar Jingshuang 16, inoculated with E20, was planted every 10th row as a control. Additionally, it was placed perpendicularly and adjacent to the test rows to ensure uniform infection spread. Disease severity (DS) for each line was assessed by averaging the percentage of leaf area covered by PM. The initial assessment was conducted approximately 6 weeks post-inoculation, when the susceptible control, Jingshuang 16, exhibited severe disease symptoms. The maximum disease severity (MDS) for each line was determined when the DS of the control reached its peak value. The field trials were maintained following standard local agronomic practices.

### Molecular genotyping, map construction, and QTL analysis

The genomic DNA was extracted using the cetyltrimethylammonium bromide (CTAB) method from young leaves of the mapping and validation populations. The RILs and parental lines were genotyped using the wheat Illumina 90K SNP array. SNPs with >20% missing data or minor allele frequency (MAF) <0.5 were excluded from further analysis. The filtered SNPs were grouped into bin markers using the BIN function of IciMapping v4.2 (Meng et al., 2015). The higher-density linkage map referenced by Wen et al. (2017) and Li et al. (2018) was constructed using the regression mapping algorithm in JoinMap v4.0. QTL mapping was analyzed using the inclusive composite interval mapping (ICIM) method in IciMapping v4.1 (Meng et al., 2015). A logarithm of odds (LOD) threshold of 2.6, determined by 1,000 permutations, was used to declare significant QTLs. The physical positions of the SNPs were according to the Chinese Spring reference genome sequence (IWGSC v1.0, https://urgi.versailles.inra.fr/blast_iwgsc/).

### Statistical analysis

Phenotypic correlation coefficients, analysis of variance (ANOVA) based on the PROC GLM model, and t-tests were performed using SAS 9.4 software (SAS Institute, Cary, NC, USA). Broad-sense heritabilities (H^2^) for PM response were calculated following the methodology described by Nyquist and Baker (1991).

### KASP marker development and validation

SNPs flanking all four QTLs were converted into KASP markers (Rasheed et al., 2016) using PolyMarker. KASP assays were conducted in a 4-μL reaction volume consisting of 2 μL of 2× KASP Master Mix, 0.045 μL of KASP primer mix, and 2 μL of genomic DNA at a concentration of 30 ng/μL. The 384-well plates were read using the PHERAstar Plus SNP detection system (BMG Labtech GmbH, Ortenberg, Germany), and genotype analysis was performed using KlusterCaller software (LGC, Hoddesdon, UK). All the KASP markers were validated using a panel of 100 Chinese cultivars.

### Identification of candidate genes for powdery mildew resistance

The selection of candidate genes was guided by the following methodologies. First, genes associated with disease resistance, pathogen response, and stress tolerance, along with SNPs located within or adjacent to the physical intervals of the QTLs, were identified using the IWGSC v1.1 reference. Second, variations within the initially screened candidate genes possessing significant mutations were selected, while those with non-significant changes were excluded. Third, genes exhibiting differential expression in leaves, stems, or spikes were selected based on data from the expVIP8 database (http://wheat-expression.com/).

## Results

### Phenotypic evaluation

The mean MDS for PM across all environments was 15.6% and 65.2% for Doumai and Shi 4185, respectively. The frequency distribution of MDS showed a continuous distribution, indicating polygenic inheritance and transgressive segregation. The MDS for PM in 262 RILs was continuously distributed at 5.6%–88.3% in Dezhou in 2021 (mean MDS 38.6%, standard error 21.1, and coefficient of variation 57.4%), 4.6%–92.3% in Dezhou in 2022 (mean MDS 38.3, standard error 22.0, and coefficient of variation 52.7%), 3.3%–83.6% in Shijiazhuang in 2021 (mean MDS 37.8%, standard error 20.0, and coefficient of variation 16.7%), and 4.3%–91.0% in Shijiazhuang in 2022 (mean MDS 38.9%, standard error 22.2, and coefficient of variation 57.0%). The MDS for the RIL population was significantly correlated (*r* = 0.55–0.76, *p* < 0.01) across all four environments. ANOVA showed that both line and line × environment interaction effects were significant for MDS (**Supplementary Figure S1**; **Table S1**). A high broad-sense heritability (*H_b_* ^2^ = 0.72) was obtained across all five environments.

### Loci for powdery mildew resistance

Among the 80,587 SNP markers in the wheat Illumina assay 90K SNP array, 10,986 SNP markers were polymorphic between the two parental cultivars. A high-density linkage map spanning 2,156.07 cM was constructed for all 21 chromosomes using 2,840 representative bin markers (Wen et al., 2017; Li et al., 2018).

**Figure 1:**
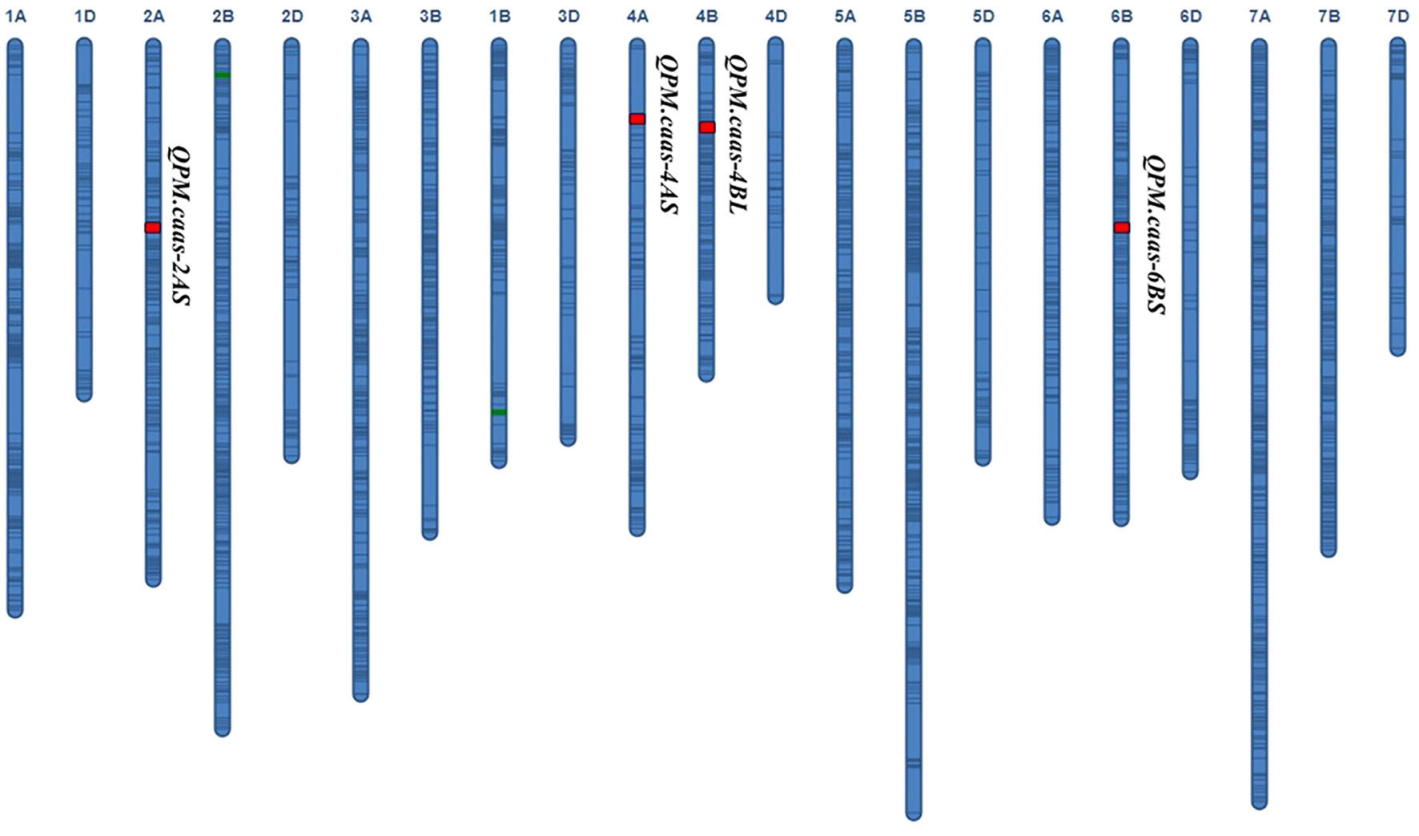
QTLs for the maximum disease severity of powdery mildew in the Doumai/Shi 4185 RIL population. QTLs, quantitative trait loci; RIL, recombinant inbred line.

Four QTLs for PM resistance were identified in two or more environments and named *QPm.caas-2AS* (*RAC875_c13116_116-RAC875_rep_c70093_1079*, 74.9–78.3 Mb), *QPm.caas-4AS* (*Tdurum_contig21233_82-Tdurum_contig10486_247*, 6.2–7.3 Mb), *QPm.caas-4BL* (*wsnp_Ra_c2711_5148302-wsnp_Ra_c10536_17322563*, 495.6–498.4 Mb), and *QPm.caas-6BS* (*RAC875_c14178_1294-RAC875_c23812_187*, 85.4–96.6 Mb) (**Table 1**; **Figure 1**). Among these, *QPm.caas-2AS* was detected in Dezhou in 2021 and Shijiazhuang in 2022, explaining 5.6%–7.6% of the phenotypic variance explained (PVE), with additive effects ranging from 3.4% to 4.0%. *QPm.caas-4AS* was detected in Shijiazhuang in 2021 and Shijiazhuang in 2022 and contributed 7.5%–8.3% of the PVE with additive effects being 4.1%–5.2%. *QPm.caas-4BL* was detected in all environments and contributed 13.8%–15.6% of the PVE, with additive effects ranging from 7.3% to 8.9%. *QPm.caas-6BS* was detected in Dezhou in 2021 and Shijiazhuang in 2021, accounting for 7.6%–9.6% of the PVE, with additive effects ranging from 5.0% to 5.2%. All the resistance alleles of *QPm.caas-2AS*, *QPm.caas-4AS*, *QPm.caas-4BL*, and *QPm.caas-6BS* were derived from Doumai.

**Table 1:** The loci identified for powdery mildew in the Doumai/Shi 4185 RIL population.

Chromosome	Environment	Interval	Position (Mb)	LOD	PVE (%)	Add
*QPM.caas-2AS*	E1, E4	*RAC875_c13116_116-RAC875_rep_c70093_1079*	74.9–78.3	3.3–3.6	5.6–7.6	3.4–4.0
*QPM.caas-4AS*	E2, E4	*Tdurum_contig21233_82-Tdurum_contig10486_247*	6.2–7.3	3.6–4.2	7.5–8.3	4.1–5.2
*QPM.caas-4BL*	E1, E2, E3, E4	*wsnp_Ra_c2711_5148302-wsnp_Ra_c10536_17322563*	495.6–498.4	10.2–11.4	13.8–15.6	7.3–8.9
*QPM.caas-6BS*	E1, E2	*RAC875_c14178_1294-RAC875_c23812_187*	85.4–96.6	4.2–4.8	7.6–9.6	5.0–5.2

RIL, recombinant inbred line; LOD, logarithm of odds; PVE, phenotypic variance explained.

### Candidate gene identification

**Figure 2:**
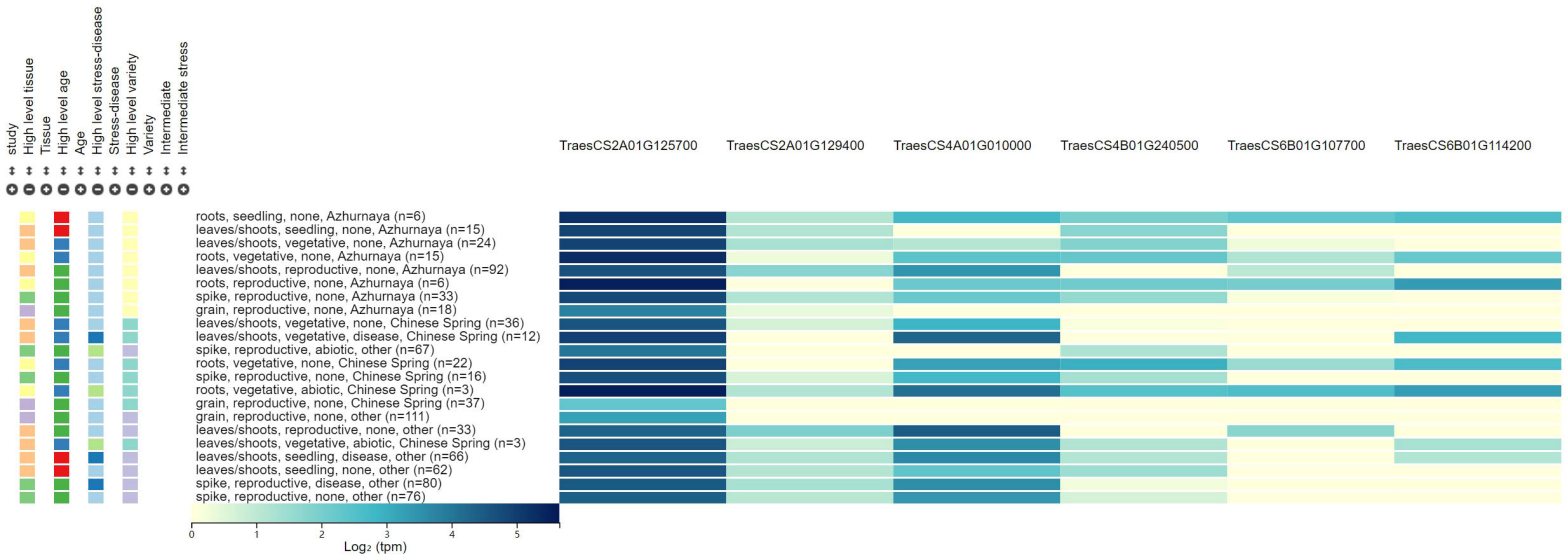
The expression patterns for the six candidate genes associated with powdery mildew resistance.

In total, six high-confidence genes were selected as the candidate genes for PM resistance. For *QPm.caas-2AS*, two candidate genes were detected. *TraesCS2A01G125700* encodes the calcium-binding and coiled-coil domain-containing protein 2. The other is *TraesCS2A01G129400*, which encodes the disease resistance protein family. *TraesCS4A01G010000* located in the genetic interval of *QPm.caas-4AS* encodes the kinase-like protein. *TraesCS4B01G240500* at *QPm.caas-4BL* encodes the calcium/calmodulin-dependent serine/threonine-kinase. For *QPm.caas-6BS*, two candidate genes were identified, i.e., *TraesCS6B01G107700* and *TraesCS6B01G114200*, which encode the NBS-LRR-like resistance protein and the disease resistance protein RPM1, respectively (**Table 2**; **Figure 2**).

**Table 2:** The candidate gene list for the powdery mildew resistance identified in the Doumai/Shi 4185 RIL population.

Candidate gene	QTL	Position (Mb)	Annotation
*TraesCS2A01G125700*	*QPM.caas-2AS*	74.2	Calcium-binding and coiled-coil domain-containing protein 2
*TraesCS2A01G129400*	*QPM.caas-2AS*	77.3	Disease resistance protein family
*TraesCS4A01G010000*	*QPM.caas-4AS*	5.8	Kinase-like protein
*TraesCS4B01G240500*	*QPM.caas-4BL*	498.9	calcium/calcium/calmodulin-dependent Serine/Threonine-kinase
*TraesCS6B01G107700*	*QPM.caas-6BS*	88.3	NBS-LRR-like resistance protein
*TraesCS6B01G114200*	*QPM.caas-6BS*	96.6	Disease resistance protein RPM1

RIL, recombinant inbred line; QTL, quantitative trait locus.

### Validation of QTLs

All four QTLs (*QPm.caas-2AS*, *QPm.caas-4AS*, *QPm.caas-4BL*, and *QPm.caas-6BS*) were used to develop available KASP markers for wheat PM resistance breeding. Although attempts were made to develop KASP markers for *QPm.caas-4AS* (*Tdurum_contig21233_82* and *Tdurum_contig10486_247*), it was non-chromosome specific or unable to be effectively distinguished between the two parental genotypes in the RIL population. Consequently, *Kasp_PMR_2AS* corresponding to *BobWhite_rep_c62964_873* (594.2 Mb) located at the genetic interval of *QPm.caas-2AS*, *Kasp_PMR_4BL* corresponding to *RFL_Contig2459_2314* (497.2 Mb) located at the genetic interval of *QPm.caas-4BL*, and *Kasp_PMR_6BS* corresponding to *RAC875_rep_c116755_285* (77.4 Mb) for *QPm.caas-6BS* were successfully developed (**Supplementary Tables S2**, **S3**). *Kasp_PMR_2AS*, *Kasp_PMR_4BL*, and *Kasp_PMR_6BS* could distinguish two genotypes in the Doumai/Shi 4185 RIL population and were used to verify their effectiveness in the 100 diverse wheat cultivars. For *Kasp_PMR_2AS*, the favorable allele (GG, accounting for 21.0%, mean MDS 13.6%) exhibited higher resistance compared to the unfavorable allele (AA, accounting for 77.0%, mean MDS 19.5%) (*p* = 0.05). For *Kasp_PMR_4BL*, the favorable allele (AA, accounting for 39.0%, mean MDS 14.3%) showed higher resistance than the unfavorable allele (GG, accounting for 43.0%, mean MDS 20.1%) at *p* = 0.05 level. For *Kasp_PMR_6BS*, the favorable allele (AA, accounting for 78%, mean MDS 15.8%) showed higher resistance than the unfavorable allele (CC, accounting for 20.0%, mean MDS 27.4%) at *p* = 0.05 level. Unlike *QPm.caas-6BS*, the resistance alleles of *QPm.caas-2AS* (accounting for 21.0%) and *QPm.caas-4BL* (accounting for 39.0%) showed lower frequency distribution than their contrasting alleles, suggesting that these resistance alleles have great potential for selection in wheat breeding for PM resistance (**Tables 2**, **3**).

**Table 3:** The development and validation of the available KASP markers for wheat powdery mildew resistance.

KASP marker	QTL	Allele	Number of lines	MDS for PM
*Kasp_PMR_2AS*	*QPm.caas-2A*	GG (favorable)	21	13.6
		AA (unfavorable)	77	19.5
*Kasp_PMR_4BL*	*QPm.caas-4B*	AA (favorable)	39	14.3
		GG (unfavorable)	43	20.6
*Kasp_PMR_6BS*	*QPm.caas-6B*	AA (favorable)	78	15.8
		CC (unfavorable)	20	27.4

KASP, kompetitive allele-specific PCR; QTL, quantitative trait locus; MDS, maximum disease severity; PM, powdery mildew.

## Discussion

PM is a severe fungal disease that threatens wheat production and results in over 300,000 tons of yield loss annually (Qie et al., 2019; Kang et al., 2020a; Wang et al., 2023). Wheat PM resistance is a typical complex quantitative inherited trait (Marone et al., 2013; Sethi et al., 2021; Saini et al., 2022). Both environment and genotype have a great impact on the incidence of PM. Until now, over 200 wheat PM resistance QTLs or genes have been identified from wheat and related species (Ullah et al., 2018; Zhang et al., 2019; He et al., 2021; Shi et al., 2021; He et al., 2022; Wang et al., 2022; Nordestgaard et al., 2021; Wei et al., 2020; Yang et al., 2017). In this study, *QPm.caas-2AS*, *QPm.caas-4AS*, *QPm.caas-4BL*, and *QPm.caas-6BS* were identified for PM resistance.

Chromosome 2A is a gene-rich region for PM resistance. Seven permanently named Pm genes, including *Pm4a-2AL*, *Pm4b-2AL*, *Pm4c-2AL*, *Pm4d-2AL*, *Pm50-2AL*, *Pm3k* (112 Mb), and *Pm52* (581.2–586.6 Mb), and 19 temporary genes or QTLs (such as *QPm.sfr-2A*, *QPminra-2A*-112, *QPm.crag-2A*, and *QPm.ttu-2A*) were reported previously (Li et al., 2019; Wu et al., 2019; Schmolke et al., 2012; Sánchez-Martín et al., 2021). The PM resistance QTL *QPm.caas-2AS.1* identified in this study (74.9–78.3 Mb) was different from *QPmbdt.nwafu-2AS*, which originated from Baidatou (111.0–174.0 Mb) (Zhou et al., 2024). Thus, *QPm.caas-2AS* may be a novel QTL. Over 15 genes or loci for PM resistance were identified on chromosome 4A and mainly located on the long arm rather than the short arm (Sun et al., 2018; Janáková et al., 2019; Liu et al., 2021), such as *Pm61*, *QPm.tut-4A* (Janáková et al., 2019), *QPm.sfr-4A.1* (Keller et al., 1999), *QPm.sfr-4A.2* (Keller et al., 1999), *Pm61* (Sun et al., 2018), and *MITW30* (Liu et al., 2021). *QPm.uga-4A* from soft red winter wheat AGS2000 was presently located between markers *tPt4753* and *wPt3515* near the centromere on chromosome arm 4AS (Hao et al., 2015). Another PM resistance QTL located on chromosome 4AS (*QPm.saas-4AS*), contributed by CM104 (Liu et al., 2021), was detected on the distal chromosome arm 4AS (2.9–4.0 Mb). In this study, *QPm.caas-4AS* was identified at 6.2–7.4 Mb of chromosome 4AS and was adjacent to *QPm.saas-4AS* originating from CM104 (Liu et al., 2021).

On chromosome 4B, 12 PM resistance genes or QTLs were previously reported (Marone et al., 2013; Kang et al., 2020a). Most loci were distributed on the 4B centromere, such as *QPm.ipk-4B* and *QPm.sfr-4B* in W7984 and *Swiss spelt* cv. *QPm.nuls-4BL* in Avocet between *XwPt1505* and *Xgwm149*; *QPm.caas-4BL.1* was located at the interval of *Xgwm149* and *Xgwm495* in Libellula (Asad et al., 2014). In addition, five loci for PM resistance were identified at 512–583 Mb of chromosome 4B by meta-analysis (Marone et al., 2013; Sethi et al., 2021; Saini et al., 2022) and different from *QPm.caas-4BL* (495.6–498.4 Mb) identified in this study. Thus, *QPm.caas-4BL* may be a novel locus for PM resistance. Chromosome 6B is another gene-rich region for PM resistance, including *Pm11* (6BS), *Pm14* (6BS), *Pm12* (6BS), *Pm20* (6BS), and *Pm54* (6BL) (Marone et al., 2013; Kang et al., 2020b; Sethi et al., 2021; Saini et al., 2022). In addition, over 12 loci (such as *QPm.umb-6BS*, *QPm.caas-6BS*, *QPm.Bgt66-6BS*, *QPm.caas-6BL.1*, and *QPm.caas-6BL.2*) for PM were identified on chromosome 6B. Meta-analyses (Marone et al., 2013; Kang et al., 2020a; Sethi et al., 2021; Saini et al., 2022) indicated that a cluster for PM resistance was identified on chromosome 6BS (67–88 Mb) and overlapped with the *QPm.caas-6BS* (85.4–96.6 Mb) identified in this study.

Powdery mildew can affect wheat yield and agronomic traits, potentially causing linkage drag, as demonstrated by some studies involving genes like *Pm8* and *Pm16*. We have previously conducted thorough multi-environment phenotypic assessments and genome-wide association analyses of agronomic traits, yield, and grain-related traits in the RIL populations of Doumai and Shi 4185 (Li et al., 2018). No co-localized associated loci were found near the four identified powdery mildew resistance QTLs, indicating that these genes do not cause linkage drag. This finding is consistent with our field observations. In the field, Doumai shows good resistance to powdery mildew and exhibits traits such as short plant height, robust stems, large grains, and large spikes.

### Six candidate genes for PM resistance were identified

Six candidate genes for PM resistance were identified, which were involved in disease resistance, redox reaction, stress tolerance, and signal transduction. These candidate genes were screened according to the following criteria: 1) the genes are located in or adjacent to the physical intervals of QTLs identified, 2) they are related to the molecular processes in pathogen response, and 3) they may be differentially expressed in leaves, stems, and spikes. Six candidate genes involved in the biological metabolism of disease resistance protein family, kinase-like protein, and NBS-LRR-like resistance protein were identified. Two candidate genes (*TraesCS2A01G125700* and *TraesCS2A01G129400*) for *QPm.caas-2AS* were identified. *TraesCS2A01G125700* encodes the calcium-binding and coiled-coil domain-containing protein 2, which can recognize pathogen effectors delivered into plant cells during the infection process, and play a crucial role in the plant’s innate immune system (Liu N. et al., 2017). *TraesCS2A01G129400* for *QPm.caas-2AS* and *TraesCS6B01G114200* for *QPm.caas-6BS* encode the disease resistance protein family, which plays a crucial role in plant innate immune system and was associated with various diseases, such as stripe rust, leaf rust, and PM reaction (Nie and Ji, 2019; Chandran et al., 2021; Li et al., 2024). *TraesCS4A01G010000* of *QPm.caas-4AS* encodes the kinase-like protein, a novel domain for wheat PM resistance gene, like *Pm13* (Li et al., 2024). *TraesCS4B01G240500* located at the genetic interval of *QPm.caas-4BL* encodes the calcium/-dependent serine/threonine-kinase, which plays important roles in a wide range of physiological functions, including plant hormone responses, metabolic regulation, and defense reactions against diseases (Chandran et al., 2021; Hu et al., 2021). *TraesCS6B01G107700*, the candidate gene for *QPm.caas-6BS*, encodes the NBS-LRR-like resistance protein, which is a crucial gene family in plant immune responses, mainly involved in the recognition and response to pathogens, and thus participates in the plant defense process (He et al., 2018, Pm21; Desiderio et al., 2021).

### Applications in wheat breeding

Although traditional breeding methods have improved PM resistance, the process is often lengthy and inefficient. KASP is a genotyping technology used for identifying SNPs and insertions/deletions (InDels). It is highly regarded for its accuracy, cost-effectiveness, and flexibility in various applications, including genomic selection, MAS in plant breeding, and molecular marker development (Rasheed et al., 2017). In this research, the markers *Kasp_PMR_2AS*, *Kasp_PMR_4BL*, and *Kasp_PMR_6BS* were developed from closely linked SNP markers and validated as effective tools for MAS in breeding programs. Furthermore, varieties with favorable alleles, PM resistance, and desirable agronomic traits, such as Lumai14, Zhongmai895, Huaimai21, Lankao24, Lumai23, Wanmai38, Yumai47, and Zhou8425B, are recommended as parent lines to enhance PM resistance in wheat breeding.

## Conclusions

In the present study, linkage analysis for PM resistance was conducted in the Doumai/Shi 4185 RIL population. Four loci on chromosomes 2A, 4A, 4B, and 6B were identified and explained 5.6%–15.6% of the phenotypic variations. Three available KASP markers (*Kasp_PMR_2AS*, *Kasp_PMR_4BL*, and *Kasp_PMR_6BS*) for PM resistance breeding were developed and validated in a natural population. The resistance loci, available KASP markers, and cultivars with more resistance alleles can be used to accelerate the progress of PM resistance breeding.

## Data Availability

The original contributions presented in the study are included in the article/**Supplementary Material**. Further inquiries can be directed to the corresponding authors.

## Supplementary material

Supplementary Figure 1 Frequency distribution of the maximum disease severity of powdery mildew in the Doumai/Shi 4185 RIL population.
