# Supplementary material for: Identification of genetic loci for powdery mildew resistance in common wheat
Source: Front Plant Sci. 2024 Oct 9;15:1443239. doi: 10.3389/fpls.2024.1443239 (PMC11496114; doi:10.3389/fpls.2024.1443239)
Supplement: Supplementary file 1 [file Table1.docx]

**Table S1** Analysis of variance of the maximum disease severity in the Doumai/Shi 4185 RIL population

| Source of variation | DF | F value ^a^ |
| --- | --- | --- |
| Environment | 3 | 1205.123*** |
| Line | 261 | 14.23*** |
| Line Environment | 780 | 3.26*** |
| Replicate (Environment) | 9 | 15.61*** |
| *H_b_^2b^* | 0.72 |  |

^a^ * indicates significance at the 0.001 probability level

^b^ *H_b_*^2^, Broad-sense heritability
