# Supplementary material for: Identification of genetic loci for powdery mildew resistance in common wheat
Source: Front Plant Sci. 2024 Oct 9;15:1443239. doi: 10.3389/fpls.2024.1443239 (PMC11496114; doi:10.3389/fpls.2024.1443239)
Supplement: Supplementary file 2 [file Table2.docx]

**Table S2** Polymorphic KASP markers for powdery mildew resistance used in this study

| **QTL** | **KASP Marker** | **Physical Position (Mb)^a^** | **Primer Name^b^** | **Sequence (5’to 3’) ^c^** |
| --- | --- | --- | --- | --- |
| *QPMR.caas-2AS* | *Kasp_PMR_2AS* | 105.1 | *Kasp_2AS_PMRA* | **GAAGGTGACCAAGTTCATGCT**TTGAATGCTGTCGAGCTACTAT |
|  |  |  | *Kasp_2AS_PMRB* | **GAAGGTCGGAGTCAACGGATT**TTGAATGCTGTCGAGCTACTAC |
|  |  |  | *Kasp_2AS_PMRC* | TGCGGTTTCTTCCCAGTTCA |
| *QPMR.caas-6BS* | *Kasp_PMR_6BS* | 77.4 | *Kasp_6BS_PMRA* | **GAAGGTGACCAAGTTCATGCT**ttggGttagatgaCacagcacaT |
|  |  |  | *Kasp_6BS_PMRB* | **GAAGGTCGGAGTCAACGGATT**ttggGttagatgaCacagcacaG |
|  |  |  | *Kasp_6BS_PMRC* | aagatacattCcttctctcaccaaT |

a Physical position (Mb) of the markers were obtained by blasting SNP flanking sequences against the Chinese Spring RefSeq v1.0 sequence (https://urgi.versailles.inra.fr/blast_iwgsc/)

b A and B indicated allele specific primer, C indicated the common reverse primer

c FAM and HEX tails used for KASP marker assays were indicated in bold
