# Supplementary material for: Identification of genetic loci for powdery mildew resistance in common wheat
Source: Front Plant Sci. 2024 Oct 9;15:1443239. doi: 10.3389/fpls.2024.1443239 (PMC11496114; doi:10.3389/fpls.2024.1443239)
Supplement: Supplementary file 3 [file Table3.docx]

**Table S3** The genotypic and phenotypic data of powdery mildew resistance for 100 wheat cultivars

| **Number** | **Name** | ***Kasp_PMR_2AS*** | ***Kasp_PMR_4BL*** | ***Kasp_PMR_6BS*** | **Maximum disease severity (%)** |
| --- | --- | --- | --- | --- | --- |
| 1 | Lumai 14 | AA | AA | AA | 5.00 |
| 2 | Zhongmai 895 | GG | GG | CC | 5.00 |
| 3 | 11CA40 | AA | GG | AA | 26.25 |
| 4 | Aca 601 | AA | GG | CC | 31.25 |
| 5 | Dorico | AA | AA | AA | 8.75 |
| 6 | Mantol | AA | AA | CC | 6.25 |
| 7 | PH82-2 | AA | AG | AA | 51.25 |
| 8 | Abbondanza | AA | GG | AA | 15.00 |
| 9 | Aifeng 3 | GG | AA | AA | 13.75 |
| 10 | Aikang 58 | GG | GG | AA | 25.00 |
| 11 | Gaoyou 503 | AA | AA | AA | 15.00 |
| 12 | Gaocheng 8901 | AA | AA | AA | 13.75 |
| 13 | Hengguan 33 | GG | GG | AA | 20.00 |
| 14 | Huapei 5 | GG | AG | AA | 7.50 |
| 15 | Huaimai 20 | AA | AA | AA | 11.25 |
| 16 | Huaimai 21 | AA | AA | CC | 8.75 |
| 17 | Jimai 19 | NN | AA | AA | 7.50 |
| 18 | Jimai 20 | AA | GG | CC | 28.75 |
| 19 | Jimai 21 | AA | AA | CC | 26.25 |
| 20 | Jimai 22 | AA | GG | AA | 15.00 |
| 21 | Jinan 13 | AA | AG | AA | 38.75 |
| 22 | Jining 16 | AA | AA | AA | 23.75 |
| 23 | Jishi02-1 | AA | GG | AA | 15.00 |
| 24 | Jinhe 9123 | GG | GG | NN | 8.75 |
| 25 | Jinmai 61 | AA | AA | AA | 28.75 |
| 26 | Lankao 24 | GG | AA | AA | 8.75 |
| 27 | Lankao 24 | GG | GG | AA | 5.00 |
| 28 | Lankao 906 | GG | AA | AA | 5.00 |
| 29 | Liangxing 66 | AA | AA | AA | 20.00 |
| 30 | Linmai 2 | AA | AA | AA | 12.50 |
| 31 | Linmai 4 | AA | AG | AA | 13.75 |
| 32 | Lumai 11 | AA | AG | AA | 18.75 |
| 33 | Luami 15 | AA | AA | AA | 8.75 |
| 34 | Lumai 21 | AA | GG | AA | 22.50 |
| 35 | Lumai 23 | AA | GG | AA | 6.25 |
| 36 | Lumai 5 | AA | GG | CC | 30.00 |
| 37 | Lumai 8 | AA | AA | AA | 18.75 |
| 38 | Lumai 9 | AA | AA | CC | 50.00 |
| 39 | Luyuan 502 | AA | GG | AA | 20.00 |
| 40 | Luomai 21 | AA | GG | CC | 43.75 |
| 41 | Neixiang188 | AA | AG | AA | 18.75 |
| 42 | Shannong 20 | AA | AA | AA | 6.25 |
| 43 | Shan 229 | AA | GG | AA | 18.75 |
| 44 | Shan 253 | GG | AA | AA | 10.00 |
| 45 | Shan 354 | AA | NN | AA | 27.50 |
| 46 | Shan 512 | AA | GG | AA | 6.25 |
| 47 | Shanmai 94 | AA | GG | AA | 12.50 |
| 48 | Shannong78-59 | AA | GG | AA | 38.75 |
| 49 | Shannong 981 | AA | AA | CC | 32.50 |
| 50 | Shanyou 225 | AA | AA | AA | 12.50 |
| 51 | Shi 4185 | GG | GG | CC | 43.75 |
| 52 | Shijiazhuang 15 | GG | AA | AA | 6.25 |
| 53 | Shijiazhuang 8 | GG | AG | AA | 7.50 |
| 54 | Shixin 828 | AA | GG | AA | 37.50 |
| 55 | Taishan 1 | AA | GG | AA | 12.50 |
| 56 | Wan 23094 | AA | GG | AA | 10.00 |
| 57 | Wanmai 29 | AA | AG | AA | 13.75 |
| 58 | Wanmai 33 | AA | AA | AA | 10.00 |
| 59 | Wanmai 38 | AA | AA | AA | 8.75 |
| 60 | Wanmai 50 | NN | GG | AA | 8.75 |
| 61 | Wanmai 52 | AA | AA | AA | 11.25 |
| 62 | Wanmai 53 | AA | GG | AA | 10.00 |
| 63 | Wennong 14 | AA | GG | AA | 26.25 |
| 64 | Wennong 5 | AA | AG | AA | 13.75 |
| 65 | Xinong 2000-7 | AA | AA | AA | 27.50 |
| 66 | Xinong 291 | AA | GG | AA | 12.50 |
| 67 | Xinong 88 | AA | NN | AA | 10.00 |
| 68 | Xiaoyan 54 | AA | AA | AA | 13.75 |
| 69 | Xiaoyan 81 | AA | GG | AA | 5.00 |
| 70 | Xinmai 9408 | AA | AA | AA | 18.75 |
| 71 | Yannong 15 | AA | GG | AA | 30.00 |
| 72 | Yannong 18 | AA | GG | CC | 13.75 |
| 73 | Yannong 19 | AA | NN | AA | 21.25 |
| 74 | Yumai 13 | AA | GG | AA | 13.75 |
| 75 | Yumai 21 | AA | GG | CC | 41.25 |
| 76 | Yumai 34 | AA | AA | AA | 10.00 |
| 77 | Yumai 35 | AA | AG | AA | 15.00 |
| 78 | Yumai 47 | AA | GG | AA | 6.25 |
| 79 | Yumai 50 | AA | GG | CC | 45.00 |
| 80 | Yumai 57 | GG | AG | NN | 15.00 |
| 81 | Yumai 63 | AA | AA | AA | 6.25 |
| 82 | Zheng 9023 | AA | GG | AA | 48.75 |
| 83 | Zhengyin 1 | GG | GG | AA | 25.00 |
| 84 | Zhong 892 | AA | GG | CC | 27.50 |
| 85 | Zhongmai 871 | GG | AA | AA | 10.00 |
| 86 | Zhongmai 875 | AA | GG | CC | 6.25 |
| 87 | Zhongyu 9 | AA | AG | CC | 66.25 |
| 88 | Zhou 8425B | AA | AA | AA | 6.25 |
| 89 | Zhoumai 11 | AA | AA | AA | 8.75 |
| 90 | Zhoumai 12 | AA | GG | CC | 13.75 |
| 91 | Zhoumai 13 | GG | AA | AA | 12.50 |
| 92 | Zhoumai 18 | GG | GG | AA | 10.00 |
| 93 | Zhoumai 22 | AA | GG | CC | 5.00 |
| 94 | Zhoumai 23 | AA | GG | AA | 12.50 |
| 95 | Zhoumai 28 | GG | AA | AA | 8.75 |
| 96 | Zhoumai 30 | AA | GG | AA | 22.50 |
| 97 | Zhoumai 31 | GG | GG | CC | 22.50 |
| 98 | Zhoumai 32 | GG | AA | AA | 16.25 |
| 99 | Zimai 12 | AA | AG | AA | 6.25 |
| 100 | Zixuan 2 | AA | AA | AA | 27.50 |
