# Supplementary figures and images for: Identification of genetic loci for powdery mildew resistance in common wheat
Source: Front Plant Sci. 2024 Oct 9;15:1443239. doi: 10.3389/fpls.2024.1443239 (PMC11496114; doi:10.3389/fpls.2024.1443239)

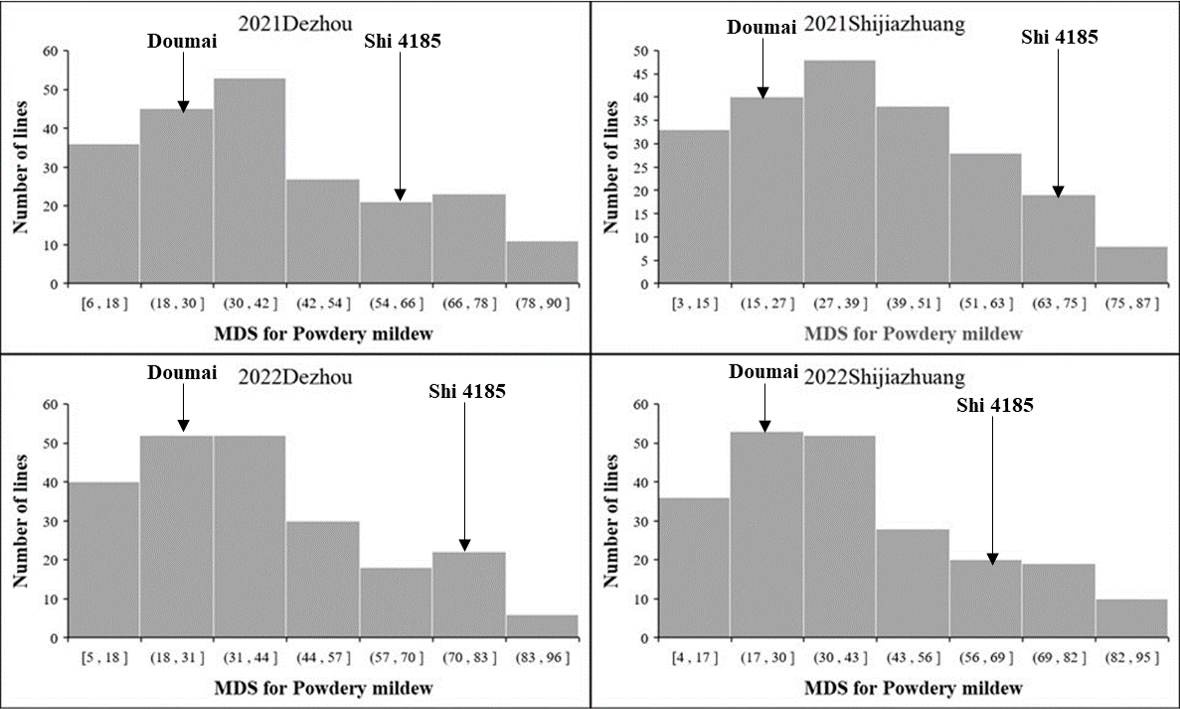

Supplement: Supplementary Figure 1 — Frequency distribution of the maximum disease severity of powdery mildew in the Doumai/Shi 4185 RIL population. [file Image1.tif]
